# Factors influencing the development and implementation of a prehabilitation program for kidney transplant candidates: A mixed-methods contextual analysis

**DOI:** 10.1016/j.ijnsa.2026.100558

**Published:** 2026-05-18

**Authors:** Avril J. Haanstra, Yvonne van der Veen, Heleen Maring, Evelien E. Quint, Adelita V. Ranchor, Stefan P. Berger, Stephan J.L. Bakker, Evelyn J. Finnema, Coby Annema

**Affiliations:** a University of Groningen, University Medical Center Groningen, Department of Health Sciences, Section of Nursing Science, Groningen 9700 RB, the Netherlands; b University of Groningen, University Medical Center Groningen, Department of Nephrology, Internal Medicine, Groningen 9700 RB, the Netherlands; c University of Groningen, University Medical Center Groningen, Department of Dietetics, Groningen 9700 RB, the Netherlands; d University of Groningen, University Medical Center Groningen, Department of Rehabilitation Medicine, Groningen 9700 RB, the Netherlands; e University of Groningen, University Medical Center Groningen, Department of Surgery, Groningen 9700 RB, the Netherlands; f University of Groningen, University Medical Center Groningen, Department of Health Sciences, Section of Health Psychology, Groningen 9700 RB, the Netherlands

**Keywords:** Kidney transplantation, Chronic kidney failure, Preoperative care, Physical fitness, Nutritional status, Psychological well-being, Person-centered care

## Abstract

**Background:**

Kidney transplantation is the preferred treatment for kidney failure. However, kidney transplant candidates often experience a decline in health while awaiting transplantation due to disease progression, comorbidities, and negatives effects of dialysis. Prehabilitation aims to optimize physical and psychological functioning prior to surgery, potentially improving overall health and outcomes. Despite its potential benefits, there are currently no official guidelines or standardized prehabilitation programs for kidney transplant candidates.

**Purpose:**

To explore factors that influence the development and implementation of a prehabilitation program for kidney transplant candidates.

**Methods:**

A mixed methods context analysis was conducted, guided by the Context and Implementation of Complex Interventions framework. Data collection included a survey, interviews, and focus groups. The survey was sent to kidney transplant candidates. It assessed current lifestyle-related practice patterns, health status including physical functioning, nutritional status, and psychological well-being, as well as needs, preferences, and perceived barriers and facilitators regarding prehabilitation. Qualitative data were collected from kidney transplant candidates and recipients, their significant others, and healthcare providers, addressing the same topics in greater depth. Quantitative data were analyzed using descriptive statistics and comparative analysis. Qualitative data were analyzed thematically.

**Results:**

Eighty-seven kidney transplant candidates completed the survey. Additionally, 22 interviews and five focus groups were conducted. Findings were that clinical consultations primarily focused on medical aspects, with less emphasis on lifestyle factors such as physical activity. Nearly all participants reported issues related to physical functioning, nutritional status, or psychological well-being, with 44% experiencing problems across all three domains. All groups expressed a strong need for prehabilitation, emphasizing that programs should be tailored to individual needs and preferences, implemented in a home-based setting, and guided by a professional. Key facilitators for engagement included peer support and professional guidance, ease of access and convenience, and high levels of intrinsic and extrinsic motivation. Identified barriers included geographical and logistical challenges, fatigue, perceived lack of need, and time constraints.

**Conclusions:**

This study highlights the need for home-based, tailored, and professionally guided prehabilitation programs for kidney transplant candidates. Key facilitators and barriers to participation were identified, emphasizing the need to address logistical, physical and psychological challenges to enhance engagement in this vulnerable population. The use of a mixed-methods approach, guided by the Context and Implementation of Complex Interventions framework, provided valuable insights into factors influencing the development and implementation of prehabilitation programs. Future research should explore the feasibility and effectiveness of prehabilitation in this vunerable patient group.

**What is already known**

 

• Kidney transplant candidates often experience physical, nutritional, psychological decline while awaiting transplantation.
• Prehabilitation may improve health outcomes prior to surgery, however, no standardized programs to optimize their health status currently exist for kidney transplant candidates.
• Barriers to implementation of prehabilitation in this population are poorly understood.

What this paper adds

• Kidney transplant candidates and recipients, their significant others, and healthcare providers report a strong need for tailored, home-based, and professionally guided prehabilitation programs addressing physical, nutritional, and psychological challenges.
• Engagement in prehabilitation is facilitated by social support, professional guidance, and accessibility, while important barriers include logistical issues, fatigue, and perceived lack of need.
• A mixed-methods approach, guided by the Context and Implementation of Complex Interventions framework, identified important factors influencing the development and implementations of a prehabilitation program for kidney transplant candidates.

Alt-text: Unlabelled box dummy alt text

## 1 Introduction

Kidney transplantation is considered the optimal treatment for individuals with kidney failure, as it is associated with a better quality of life, well-being, and survival benefits compared with dialysis (Hellemans et al., 2020; Jansz et al., 2018; Watschinger et al., 2019). Worldwide, 110 467 kidney transplants were performed in 2024 (Global Observatory on Donation and Transplantation 2024). Although the number of kidney transplants is increasing each year, individuals eligible for transplantation are placed on a waiting list due to a continuous shortage of suitable kidney donors. Within the Eurotransplant region, comprising eight European countries, 9953 individuals were on the kidney transplant waiting list in 2025 (Eurotransplant, 2025). In the Netherlands, approximately 18,000 people with kidney failure received renal replacement therapy, of whom 12,843 underwent kidney transplantation in 2024 (Nefrovisie, 2025). Additionally, at the end of 2025, there were 3493 individuals on the kidney transplant waitlist, representing a 2.8% increase compared to 2024 (Dutch Transplant Foundation, 2025a). The median waiting time in the Netherlands is approximately 12 months for a kidney from a living donor and approximately 22 months for a kidney from a deceased donor (Dutch Transplant Foundation, 2025b).

While awaiting kidney transplantation, candidates often experience deteriorating health due to disease progression, comorbidities, and the negative effects of dialysis treatment (Francis et al., 2024; Hernandez et al., 2015; Webster et al., 2017). This deterioration is associated with impaired physical fitness, poor nutritional status, and psychological problems, which can lead to a lower quality of life (Johansen et al., 2017; Kang et al., 2017; Kirkman et al., 2021; Li et al., 2023). As kidney function declines, waste products and excess fluid accumulate in the body, resulting in symptoms such as fatigue, muscle wasting, weakness, loss of appetite, and fluid retention. Furthermore, disease progression can lead to complications such as anemia, bone disease, and electrolyte imbalances, which continues to adversely affect the health status of kidney transplant candidates (Thomas et al., 2008; Webster et al., 2017). Additionally, multiple studies have shown that diabetes, hypertension, and cardiovascular disease are the most prominent co-existing conditions in the kidney transplant candidate population (Cha and Han, 2020; Matsushita et al., 2022; Prichard, 2000; Webster et al., 2017). Moreover, while dialysis is a lifesaving treatment option, it can lead to symptoms such as hypotension, muscle cramps, infections, and vascular access-related issues (Thomas et al., 2008). This overall poor health status of kidney transplant candidates often results in a generally low level of physical activity and poor nutritional status, inducing a cycle of decreased physical fitness, involving multiple factors, including protein-energy wasting, muscle loss, malnutrition, micronutrient deficiencies, inflammation, and fatigue (Zelle et al., 2017), which in turn can lead to cognitive and psychological problems (Kittiskulnam et al., 2016). Overall, kidney transplant candidates face significant health challenges while awaiting kidney transplantation, highlighting the vulnerability of this population.

The waitlist period provides a unique opportunity to implement a pre-transplant intervention to improve the health status of kidney transplant candidates. Prehabilitation involves optimizing an individual’s physical and psychological condition prior to surgery in order to withstand the stress associated with the procedure (Minnella and Carli, 2018), and may serve as an effective intervention for improving the health status of kidney transplant candidates. The main elements of a multimodal prehabilitation program include physical training, dietary management, and psychological interventions (Minnella and Carli, 2018). In other study populations, including surgical and cancer patients, prehabilitation has shown to enhance postoperative recovery time and improve outcomes, including surgical complications, length of hospitalization, and quality of life (Barberan-Garcia et al., 2018; Cabilan et al., 2015; Myers and Fonda, 2016; Tsimopoulou et al., 2015). The effect of prehabilitation in kidney transplant candidates has been assessed in a limited number of studies (Gross et al., 2017; Lorenz et al., 2020; Ma et al., 2022; McAdams-DeMarco et al., 2019). While these studies showed that prehabilitation increased physical activity and improved fatigue, as well as improving walking time and hand grip strength in kidney transplant candidates during the waitlist period, they primarily focused on individual components of prehabilitation rather than a comprehensive approach.

Currently, no official guidelines or standardized programs exist for prehabilitation in kidney transplant candidates. Implementing a multimodal prehabilitation program poses challenges due to the unpredictable timing of transplantation, disparities in candidates’ health status, and variation in dialysis schedules. To address these challenges and effectively implement prehabilitation, a thorough understanding of current practices, candidates’ physical, nutritional, and psychological status, needs and preferences, potential barriers and facilitators, and suitable implementation strategies is essential.

This study aimed to explore factors that can potentially influence the development and implementation of a prehabilitation program for kidney transplant candidates. The objectives included examining current lifestyle-related practice patterns for kidney transplant candidates, assessing the health status of this population in terms of physical functioning, nutritional status, and psychological well-being, identifying needs and preferences regarding prehabilitation, and exploring potential barriers and facilitators to prehabilitation among kidney transplant candidates and healthcare providers involved in their care.

## 2 Methods

### 2.1 Study design and setting

**Fig. 1:**
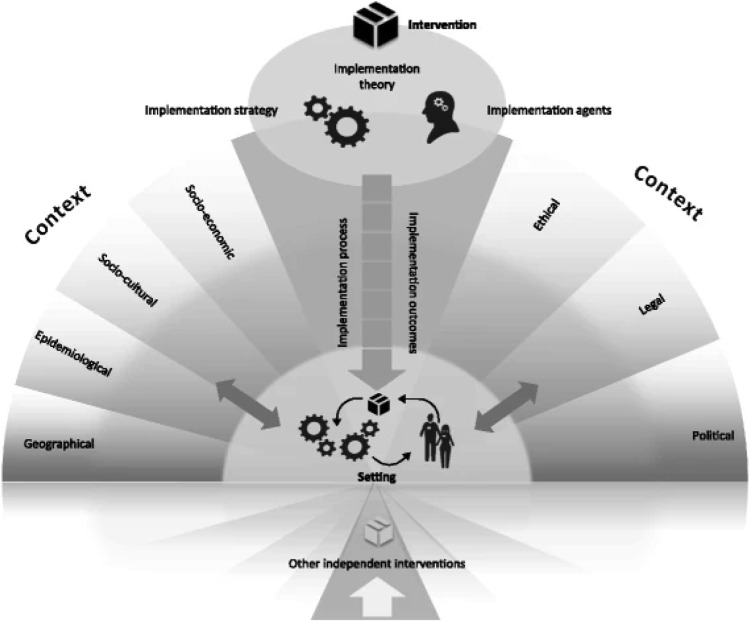
The Context and Implementation of Complex Interventions framework (Pfadenhauer et al., 2017).

We performed a mixed-methods context analysis incorporating a survey, sequential semi-structured interviews, and focus groups, guided by the Context and Implementation of Complex Interventions framework (Fig. 1) (Pfadenhauer et al., 2017). This framework provides a structured approach to conceptualize, describe, and understand the multiple influences on the development and implementation of a prehabilitation program. It builds on existing frameworks by adding a macro-level perspective to capture all aspects of complex interventions, and facilitates a structured and comprehensive assessment of development and implementation factors. This assessment considers various contextual domains, including geographical, epidemiological, and socio-cultural influences, as well as implementation aspects, and intervention setting (Pfadenhauer et al., 2017). The application of the Context and Implementation of Complex Interventions framework in this study is summarized in Table 1.

**Table 1:** Application context and implementation of complex interventions framework and integration of quantitative and qualitative data in this study.

CICI Component	Description	Application in This Study	
		Quantitative data	Qualitative data
Setting	Refers to the specific setting in which the intervention is put into practise	Survey among kidney transplant candidates: questions about current practice patterns	Interviews and focus groups with patients and healthcare providers: explored how prehabilitation could be integrated in current practice
Context domains	Geographical, epidemiological, sociocultural, and socio-economic factors of influence on development and implementation on prehabilitation	Survey among kidney transplant candidates: questions about (e.g.) health status, motivation, cultural background, socioeconomic status	Interviews and focus groups with patients, significant others, and healthcare providers: explored more in-depth which factors regarding (e.g.) health status, motivation, social support, access to care, were relevant for prehabilitation in kidney transplant candidates
Implementation domains	Factors related to intervention development and implementation process, strategies and agents	Survey among kidney transplant candidates: questions about preferences regarding the design of (content, timing, roles, and mode of delivery) and barriers and facilitators for prehabilitation	Interviews and focus groups with patients and healthcare providers: explored preferences regarding the design of a prehabilitation program, barriers and facilitators for the implementation of prehabilitation, and possible implementation strategies

The study was conducted in the University Medical Center Groningen, the Netherlands. It was approved by the Medical Ethical Committee of the University Medical Center Groningen (METc 2021/461). All participants provided written or verbal consent before participating in the study. Data were coded and processed anonymously.

### 2.2 Quantitative methods

#### 2.2.1 Study population

Individuals aged 18 years or older who were on the kidney transplant waiting list of the University Medical Center Groningen in November 2021 were invited to participate. Exclusion criteria included inability to read or speak Dutch, and being waitlisted for a combined organ transplantation (e.g., pancreas-kidney).

#### 2.2.2 Data collection and procedures

Current lifestyle-related practice patterns, health status, needs and preferences, and potential barriers and facilitators regarding prehabilitation were assessed through a self-reported survey guided by the Context and Implementation of Complex Interventions framework.

The survey was sent to eligible individuals (*n* = 138) by post with prepaid return envelopes. A reminder was sent to non-responders after two weeks.

#### 2.2.3 Socio-demographic and clinical characteristics

Socio-demographic information of kidney transplant candidates was collected by a self-report questionnaire and comprised age (in years), gender (male/female), marital status (partner/no partner), nationality (Dutch/non-Dutch), economic status (short on money every month/break even every month/saving money every month/declined to answer), educational level (primary/secondary/university) and working status (employed/(pre)retired/incapacitated/other).

Data regarding dialysis were obtained from the University Medical Center Groningen kidney transplant waitlist, and consisted of type of dialysis (no/hemodialysis/peritoneal dialysis) and dialysis period (in months), and time on the waitlist for kidney transplant (in months). Comorbidities were assessed using a checklist of eleven common medical conditions, adapted from the General Health Survey used by the Central Office for Statistics in the Netherlands (Arnold et al., 2004; Kempen et al., 1997). Respondents were asked to indicate whether they had been diagnosed with either of the listed conditions. If a diagnosed medical condition was not included in the list, they could report it in an additional open-ended question.

#### 2.2.4 Lifestyle-related practice patterns

Current lifestyle-related practice patterns were evaluated by means of thirteen items, several of which included multiple questions. The items addressed physical activity, diet, and stress management, as well as whether kidney transplant candidates received advice from healthcare providers and were offered programs to support these areas.

#### 2.2.5 Health status

Physical functioning was assessed using the Duke Activity Status Index (DASI) questionnaire and two single-item measures: the Dutch Physical Activity guidelines and the Dutch Fitnorm. The *DASI* assesses functional capacity through twelve items that address various aspects of functioning, each answered dichotomously (yes/no). The total score ranges from 0 to 58.2, with a score of ≤ 34 indicating low functional capacity. The DASI has demonstrated reliability in measuring exercise capacity in individuals with kidney failure and is considered a valid measure of pre-operative fitness (Mathiowetz et al., 1984; Ravani et al., 2012). The *Dutch Physical Activity guidelines* assesses moderate-intensity physical activity through a single item on the frequency of engaging in at least 30 min of such activity per week, with answers ranging from “less than once” to “five times or more”. Respondents meet the guideline if they engage in moderate-intensity activity for at least 30 min on five or more days per week (Gezondheidsraad, 2017). The *Dutch Fitnorm* assesses high-intensity physical activity with a single item regarding the frequency of engaging in at least 20 min of high-intensity physical activity per week to strengthen bones and muscles. Response options range from “less than one” to “three times or more”. Meeting the standard requires activity at least three times per week (Gezondheidsraad, 2017).

Nutritional status was evaluated by the Patient-Generated Subjective Global Assessment Short Form (PG-SGA-SF) and body mass index (BMI). The *PG-SGA-SF* is a self-report questionnaire assessing nutritional status across four domains: changes in body weight, changes in nutritional intake over the past month, experiences of nutrition-related symptoms in the preceding two weeks, and changes in activities and functions over the past month. A score of ≥4 indicates an increased risk of malnutrition. The questionnaire is widely recognized for its high validity (Abbott et al., 2016; Kosters et al., 2020) and reliability (Kellett et al., 2016). Weight and height were obtained from the PG-SGA-SF, and *BMI* was calculated by dividing weight (in kilograms) by height (in meters) squared (kg/m^2^). A BMI score of >27.5 was used to indicate overweight, while a score of <20 indicated underweight (Di Angelantonio et al., 2016).

Psychological well-being was assessed using the subjective fatigue subscale of the Checklist Individual Strength (CIS-8R), the General Anxiety Disorder questionnaire (GAD-7), and the Patient Health Questionnaire (PHQ-9). Fatigue was assessed using the *CIS-8*R, which includes eight statements related to feelings of fatigue rated on a 7-point Likert scale from 1 (yes, totally agree) to 7 (no, totally disagree). A score of ≥27 indicated abnormal fatigue (Bültmann et al., 2000). The subjective fatigue subscale demonstrates strong validity, with a reliability coefficient (α) of 0.96 (De Vries et al., 2003). In this study, Cronbach’s alpha was 0.91. The *GAD-7* assesses symptoms of anxiety through seven items rated on a 4-point Likert scale (0 = not at all; 4 = almost every day), with total scores ranging from 0 to 21. Higher scores indicated more severe symptoms of anxiety. A cut-off score of ≥5 was used to identify clinically relevant cases. The scale has shown to have good internal reliability and validity (Spitzer et al., 2006), with Cronbach’s alpha of 0.88 in this study. Symptoms of depression were assessed using the *PHQ-9*, which includes nine items rated on a 4-point Likert scale (0 = not at all; 3 = almost every day), with total scores ranging from 0 to 27. Higher scores indicate more severe symptoms of depression, with a score of ≥10 indicating moderate depressive symptoms. The PHQ-9 is shown to be a valid and reliable measurement of depressive symptoms (Kroenke et al., 2001), with Cronbach’s alpha of 0.76 in this study.

#### 2.2.6 Needs and preferences prehabilitation

The needs of kidney transplant candidates for prehabilitation were assessed by two items. The first item consisted of questions regarding the need to improve strength and aerobic capacity, optimize nutritional status, and manage stress, with responses categorized as “yes”, “no”, or “not applicable”. The second item was an open-ended question: “Can you explain why you do or do not need this support?”. Similar responses were grouped into categories.

The preferences of kidney transplant candidates for the prehabilitation program were assessed using eleven statements addressing program design, including preferences for individual, group or combined participation, and guided or unguided participation. Responses were categorized as “yes”, “no”, or “not applicable”. The candidates could select multiple preferences for related questions, such as participating individually and in a combined individual/group setting.

#### 2.2.7 Potential barriers and facilitators

To explore what motivates kidney transplant candidates to work on their overall health, two open-ended questions were used: “Are you currently working on your overall health? If yes, how?” and “What are the three most important reasons for you to work on your overall health?”. Barriers preventing candidates from working on their overall health were assessed with a single open-ended question: “What is currently holding you back from working on your overall health?”.

### 2.3 Qualitative methods

#### 2.3.1 Study population

The study population consisted of kidney transplant candidates and recipients, as well as their significant others. It also included healthcare providers involved in their regular care such as nephrologists, dieticians, nurse practitioners, medical social workers, and nurses. Although physiotherapy was not involved in the regular care for kidney transplant candidates, a physiotherapist was included to provide expert insights given the central role of physical functioning in prehabilitation. Inclusion criteria encompassed individuals aged 18 years or older who were waitlisted for kidney transplantation or had undergone kidney transplantation within the past two years at the University Medical Center Groningen, as well as significant others of kidney transplant candidates or recipients aged 18 years or older, and healthcare providers from hospitals and dialysis centers in the northern region of the Netherlands. Exclusion criteria included inability to speak Dutch and being waitlisted for or having previously undergone a combined organ transplantation (e.g., pancreas-kidney).

#### 2.3.2 Data collection and procedures

Semi-structured interviews and focus groups were conducted to obtain in-depth insights into current lifestyle-related practice patterns of kidney transplant candidates, their health status, and needs and preferences, and potential barriers and facilitators regarding prehabilitation. Background information, including age, marital status, and clinical aspects, such as time of diagnosis and dialysis status, was collected in conjunction with the assessment of the DASI unless the data had already been provided in the survey for participants identified as kidney transplant candidates.

Data were collected between October 2021 and April 2022. Kidney transplant candidates could indicate interest in participating in an interview and/or a focus group after completing the survey to provide additional in-depth information. Kidney transplant recipients were invited via an announcement in the University Medical Center Groningen Transplant Center newsletter for individuals with organ failure and through referrals from nephrologists. Additionally, significant others of individuals with kidney failure were invited through referrals from participants or their healthcare providers. Nephrologists were invited via email from the Head of Internal Medicine at the University Medical Center Groningen. Moreover, healthcare providers were invited through referrals from participating nephrologists.

The semi-structured interview and focus group guides (see supplementary file) were developed collaboratively by the first author (A.H.) and researcher C.A. and was subsequently reviewed by the research team. Guide development was guided by the Context and Implementation of Complex Interventions framework and employed an inductive approach. Due to COVID-19 restrictions, the majority of the interviews and focus groups were conducted online via Microsoft Teams, with one interview conducted in person at the University Medical Center Groningen. The first author conducted the majority of the interviews, while C.A. conducted three interviews with nephrologists. C.A. and A.H. led the focus groups. All sessions were audio recorded and transcribed verbatim in Microsoft Word 365 by researchers A.H, Y.V, and E.Q. The transcripts were returned to participants for review and approval.

### 2.4 Data analysis

#### 2.4.1 Quantitative data analysis

Survey data were analyzed using the appropriate descriptive statistics based on normality of data, including means, standard deviations (SDs), medians, interquartile ranges (IQRs), and frequencies (n/%). To compare socio-demographic and clinical characteristics between the study population and the total number of kidney transplant candidates to whom the survey was sent, either the Mann-Whitney U test or the independent T-test was used for continuous variables, depending on normality assumptions. For categorical variables, the Chi-square test was used. *P*-value was set at <0.05. Statistical analyses were performed using SPSS software version 28.

#### 2.4.2 Qualitative data analysis

To explore in-depth individual experiences, the thematic analysis method by Clarke and Braun (2014) was used to identify recurring themes in participants’ experiences. The transcripts were uploaded in ATLAS.ti version 24.1, and A.H. and C.A. performed the data analysis. Familiarization of the data involved reading the transcripts and reviewing observational notes. Initially, A.H. and C.A. independently coded the data using an abductive approach. After the first five interviews, the researchers compared and refined the codes, and developed a codebook. Remaining interviews and focus groups were coded, with any unclear codes discussed and adjusted as needed. Themes and sub-themes were developed by grouping related codes based on shared characteristics or underlying meanings. The final step involved refining the themes and assigning clear labels.

## 3 Results

### 3.1 General characteristics

#### 3.1.1 Survey

Of the 138 eligible kidney transplant candidates, 87 completed the survey (response rate 63%). The median age of the respondents was 61 years (IQR 54–75). Most of the respondents were male (69%), had a partner (77%), had completed secondary education (44%), and held Dutch nationality (93%). Nearly 60% of the respondents underwent dialysis, with the majority undergoing hemodialysis (73%). The median duration of dialysis was 12 h (IQR 12–30) per week. The majority of the respondents reported having one comorbidity (28%), followed by two (25%) or no other comorbidities (25%).

Table 2 shows the demographic and clinical characteristics of kidney transplant candidates who completed the survey, compared to the total number of candidates to whom the survey was sent. Only age showed to have a statistically significant difference (*p* = 0.03) between the groups, with a higher age for individuals who completed the survey.

**Table 2:** Demographic and clinical characteristics kidney transplant candidates.

	Survey (*n* = 87)	Total (*n* = 138)	*P*-value
Age, years (median, IQR)	61.0 (54–75)	59.5 (51.8–66)	**0.03**
Sex (n, % male)	60 (69.0)	93 (67.4)	0.61
Partner (n, % yes)	67 (77.0)		
Educational level (n, %)			
• Primary	28 (32.2)		
• Secondary	38 (43.7)		
• University	21 (24.1)		
Nationality (n, % Dutch)	81 (93.1)		
Employment status (n, %)			
• Paid work	28 (32.2)		
• Incapacitated/unemployed	25 (28.7)		
• (pre)Retired	25 (28.7)		
• Other	9 (10.3)		
Economic status (n, %)			
• Short on money every month	7 (8.0)		
• Break even	15 (17.2)		
• Saving money every month	51 (58.6)		
• Does not wish to answer	13 (14.9)		
• missing	1 (1.1)		
Comorbidities (n, % yes)	65 (74.7)		
Dialysis (n, % yes)	52 (59.8)	85 (61.6)	0.57
• Hemodialysis	38 (43.7)	65 (47.1)	0.55
• Peritoneal	14 (16.1)	20 (14.5)	
Dialysis period, months (median, IQR)	15.6 (7.5–24.8)	15.9 (8.2–26.8)	0.09
Waitlist period, months (median, IQR)	26.9 (14.8–41.4)	26.3 (15.0–40.8)	0.83

Note. Educational level, nationality, employment status, economic status, and comorbidities were not available for the total number of kidney transplant candidates on the waitlist.

#### 3.1.2 Interviews and focus groups individuals with kidney failure

Individual interviews were conducted with six kidney transplant candidates, and eight kidney transplant recipients. In addition, eight candidates participated in two focus groups, consisting of three and five participants. The median age of the candidates was 63 (53.8–74.8) years, whereas the recipients had a median age of 58 (47.0–64.3) years. The majority of the participants were male and had a partner. Educational levels varied between groups, with higher education more common among interview participants. The majority of recipients were employed at the time of the interview, whereas the majority of candidates were incapacitated, unemployed, or (pre)retired. Half of the candidates in the focus groups, and recipients before transplantation, received dialysis, while the majority of the candidates in the interviews were on dialysis. An overview of the demographic and clinical characteristics of the three groups are shown in Table 3.

**Table 3:** Demographic and clinical characteristics kidney failure population.

	KTCs (interviews) (*n* = 6)	KTCs (focus groups) (*n* = 8)	KTRs (interviews) (*n* = 8)
Age, years (median, IQR)	63 (53.8–74.8)	61 (56.3–68.5)	58 (47.0–64.3)
Sex (n, % male)	5 (83.3)	5 (62.5)	5 (62.5)
Partner (n, % yes)	5 (83.3)	4 (50.0)	6 (75.0)
Educational level (n, %)			
** •** Primary	1 (16.7)	4 (50.0)	0 (0.0)
** •** Secondary	0 (0.0)	2 (25.0)	3 (37.5)
** •** University	5 (83.3)	2 (25.0)	5 (62.5)
Nationality (n, % Dutch)	6 (100.0)	8 (100.0)	8 (100.0)
Employment status (n, %)			
** •** Paid work	1 (16.7)	1 (12.5)	4 (50.0)
** •** Incapacitated/unemployed	3 (50.0)	4 (50.0)	2 (25.0)
** •** (pre)Retired	2 (33.3)	3 (37.5)	1 (12.5)
** •** Other	0 (0.0)	0 (0.0)	0 (0.0)
Dialysis (n, % yes)	6 (100.0)	4 (50.0)	4 (50.0)^a^
Dialysis period, months (median, IQR)	19.0 (12.1–35.0)	16.5 (6.0–22.5)	13.0 (3.1–22.5)^a^
Time post-transplant (months)			15 (11.0–18.0)
Duration interview/focus group, min (mean, SD)	33.9 (6.4)	95.6 (31.5)	59.6 (11.0)

Note. KTCs: kidney transplant candidates; KTRs: kidney transplant recipients.

a Before transplant.

#### 3.1.3 Focus group significant others

Three significant others (two partners and one parent) of individuals with kidney failure participated in a single focus group. All participants were male and held Dutch nationality. The duration of the focus group was 74 min.

#### 3.1.4 Interviews and focus groups healthcare providers

Eight nephrologists from seven different hospitals or dialysis centers in the northern Netherlands were interviewed. The mean duration of these interviews was 38.7 ± 7.4 min. The nephrologists had a mean of 12.5 ± 5.9 years of professional experience.

Fourteen healthcare providers participated in two focus groups, including four nurse practitioners, four dieticians, three medical social workers, a kidney failure nurse, a dialysis nurse and a physiotherapist. The participants worked at six different hospitals or dialysis centers in the Northern region of the Netherlands and were involved in the regular care of kidney transplant candidates. The mean duration of the sessions was 77.9 ± 2.9 min.

### 3.2 Current practice patterns lifestyle factors

Findings from the survey showed that 61% of kidney transplant candidates received advice or recommendations related to physical activity, dietary management, and/or stress management as part of current practice. The predominant recommendations offered were for dietary management mainly focusing on dietary restrictions related to kidney failure (53%), followed by stress management (30%). Advice on physical activity was provided to 15% of the candidates, mainly by their medical specialist or nurse practitioner involved in their regular care.

These results are consistent with the findings from the interviews and focus groups. Kidney transplant candidates indicated to have appointments with their nephrologist or nurse practitioner every three months, depending on their health status and disease progression. Overall, these appointments have a strong medical focus on kidney function and laboratory values, with less emphasis on lifestyle factors such as physical activity, largely due to time constraints and lack of exercise guidelines.

*“… You have a fifteen minutes appointment in which you need to ask how things are going and check the medication. And discuss the lab results, fill out a new lab request and schedule the next appointment. Yes, then you are left with just two minutes in a packed consultation to quickly mention that it would be better to exercise more.” (Nephrologist, interview 9)*

Additionally, kidney transplant candidates usually have one to three consultations with a dietician and a medical social worker, depending on the candidate’s circumstances. The dietician and medical social worker are often involved in multidisciplinary meetings concerning the management of the candidate. A physiotherapist is generally not involved in the care of kidney transplant candidates.

The majority of the participants reported that no structured programs were offered to kidney transplant candidates regarding physical activity, dietary management and/or stress management. A small number of individuals with kidney failure participated in the STERK program, aimed at enhancing self-management, autonomy, and personal strength in managing health or a combined lifestyle intervention (Wiegersma et al., 2021). However, the majority of the individuals and healthcare providers indicated that both programs were not consistently implemented in practice due to factors such as candidate eligibility, implementation restrictions, and financial burdens.

*“We have the STERK program… But to be honest, I find it quite difficult to keep running it consistently, let’s put it that way. Because when you want to start something like that, the question always comes up, who qualifies for it? And then it becomes quite challenging to figure that out …” (Nephrologist, interview 11)*

In addition, although the majority of the dialysis centers offered cycling on specially designed bicycles for candidates during their dialysis sessions, this was not typically considered a formal program by kidney transplant candidates and recipients, and healthcare providers, as it usually lacked structured guidance and inconsistent implementation.

*“… Often, I help them lose weight for the transplant. That’s often a goal and it’s also quite helpful. But beyond that, we don’t have a specific protocol or a special program for people on the waiting list.” (Dietician, focus group 4)*

### 3.3 Health status kidney transplant candidates

#### 3.3.1 Physical functioning, nutritional status and psychological well-being

**Fig. 2:**
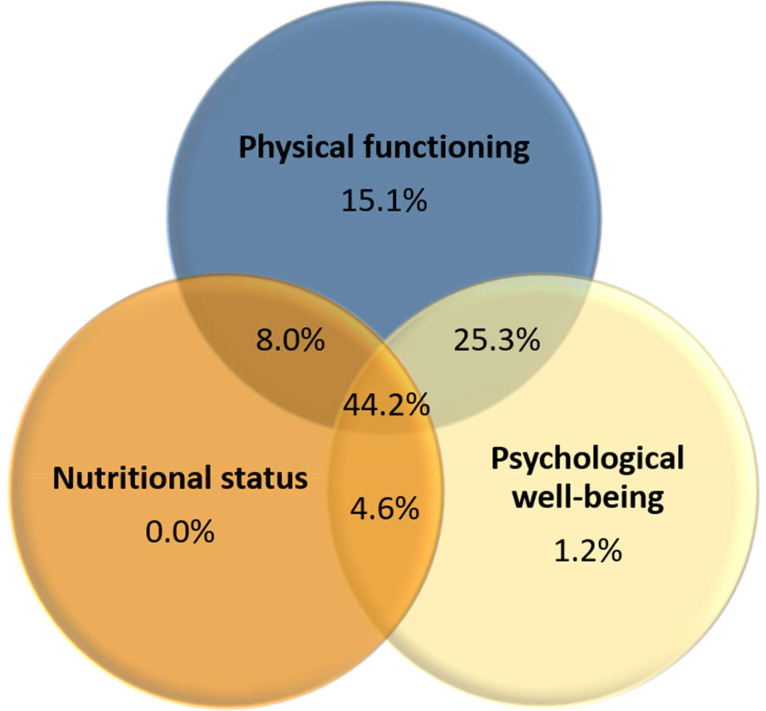
Venn-diagram with percentages of kidney transplant candidates experiencing problems in each domain.

The survey data showed that nearly all kidney transplant candidates (99%) experienced one or more problems in physical functioning, nutritional status, or psychological well-being, with 44% experiencing problems in all three domains (see Fig. 2). Of the candidates that experienced problems in two out of the three domains, the majority (25%) reported problems in physical functioning and psychological well-being.

Regarding physical functioning, 93% of the kidney transplant candidates experienced problems in the physical domain, with almost 75% experiencing two or three problems. Of the candidates, 46% had a DASI score <34, indicating low functional capacity. Additionally, the majority of the candidates did not engage in at least 30 min of moderate-intensity physical activity five days a week (77%) or in at least 20 min of high-intensity physical activity three times a week (71%). Regarding nutritional status, more than half of the candidates (56%) experienced one or more nutritional problems. The mean BMI of the kidney transplant candidates was 26.1 ± 3.4 kg/m^2^, with 32% classified as overweight and 1% as underweight. Additionally, 35% of the candidates had a PG-SGA-SF score of >4, indicating a risk of malnutrition. Regarding psychological well-being, almost half of the candidates reported one problem, while 28% reported two or three problems. Specifically, 74% experienced a high level of fatigue, 25% had mild to severe symptoms of anxiety, and 36% had mild to moderate-severe symptoms of depression.

These results were supported by the qualitative findings which identified several key factors influencing the overall health of kidney transplant candidates. The majority of the participants reported a noticeable decline, particularly in strength and endurance. Participants frequently described that daily activities required more effort, which they attributed to the progression of kidney failure, the negative effects of dialysis, and the impact of comorbidities.

*“…I still manage my household myself, but I realize that I really need to break it down into smaller tasks. ... I’ve always lived in a single-family home, and I could clean the entire house in a day, but I can't even do that with my apartment now. So, I do notice that my fitness level has deteriorated." (Kidney transplant candidate 3, focus group 1)*

Various factors influenced participants’ nutritional status. While some participants reported having a stable weight, others experienced weight fluctuations primarily due to fluid retention related to dialysis. Additionally, participants revealed struggles with being overweight, and variability in appetite was noted as well, with some participants experiencing a reduced appetite, while others described having a good appetite.

*“I have always been overweight throughout my life, and in order to be on the waitlist, I had to lose at least eight kilos. But despite that, I am still far too heavy, so I really have to be very careful about it.” (Kidney transplant candidate 3, focus group 1)*

Emotional adaptation and optimism were frequently mentioned as key factors influencing psychological well-being among kidney transplant candidates and recipients. However, participants highlighted as well the negative impact of disease progression and dialysis, and the uncertainty associated with being on the waitlist, particularly not knowing when they might receive a kidney from a deceased donor.

*“I am now on the transplant list, but I have no idea when it will happen. It feels like a big black hole, and that makes it so unpredictable. … The uncertainty makes it so unpredictable, at least that's how I experience it, and I find that very difficult.” (Kidney transplant candidate 4, focus group 3)*

Additionally, fatigue was frequently mentioned, with participants describing overall fatigue, fatigue after exertion, or varying levels of fatigue throughout the day.

The key factors mentioned influencing the overall health of kidney transplant candidates were consistent with those identified by healthcare providers and significant others of candidates and recipients, particularly concerning the decline in strength and endurance, as well as high levels of overall fatigue.

### 3.4 Needs and preferences prehabilitation

The survey data showed that 79% of kidney transplant candidates expressed a need for a prehabilitation program. The majority reported they would like to improve their strength and endurance (73%), followed by optimizing nutritional status (48%), and learning to cope with stress (30%). Overall, 22% of the candidates indicated the need for a prehabilitation program including all three domains.

Most candidates (64%) expressed a preference for an individual program, while 35% indicated interest in a combined group and individual approach. Moreover, they favored a program tailored to their needs rather than a standard program for everyone. Fifty-eight percent preferred supervision from a professional, whereas 33% opted for a program with no supervision. A majority (66%) indicated a preference for following the program at home, although 26% of the kidney transplant candidates indicated they would like to follow a program both at home and in a hospital setting. Moreover, candidates receiving dialysis preferred non-dialysis days (58%) over dialysis days (34%) for scheduling program activities.

The qualitative findings supported the quantitative results, as the majority of the candidates and recipients, their significant others, and healthcare providers emphasized the importance of a prehabilitation program, with physical functioning identified as most important component. Participants emphasized the need to address the connection between the three domains: physical functioning, nutritional status, and psychological well-being. However, nutritional status was generally considered less important in a prehabilitation program, often because participants were already following a specific diet. In the survey, participants were asked about their perceived need regarding stress management rather than specifically addressing fatigue. However, in the interviews and focus groups, dealing with fatigue emerged as another key factor. Furthermore, participants emphasized the value of peer support, the need for clear, practical information and advice, and a better understanding of available exercise options. There was also a focus on promoting self-management, and addressing challenges related to motivation.

Qualitative findings identified diverse preferences among candidates and recipients, their significant others, and healthcare providers regarding the structure of the prehabilitation program, with the majority aligning with the quantitative results. Some participants expressed a desire for an individual program, while others preferred a combined approach, incorporating both individual and group elements. A hybrid program was also suggested as a potential option. The majority of the participants indicated a preference for a program tailored to kidney transplant candidates’ specific needs and capabilities.

*“I think it is very good if things are offered in a variety of ways… I think you should offer a wide range of things because we are all different and are all on different (health)levels.” (Kidney transplant candidate 3, focus group 3)*

Guidance was consistently reported by kidney transplant candidates and recipients as crucial, particularly for physical activities, and participants emphasized the importance of receiving support from someone with relevant expertise. This perspective was shared among their significant others and healthcare providers.

*“Guidance is incredibly important. It’s not enough to just hand out an information package or give tips. Changing lifestyle habits is extremely difficult, especially making them stick. You really need guidance for that.” (Significant other 4, focus group 2)*

Some participants preferred to follow the program within their home environment, with some specifying a preference for home-based exercises. Monitoring progress was considered essential. Participant’s preferences for the timing of the program varied, some preferred non-dialysis days, while others favored engaging before or during dialysis sessions. Healthcare providers emphasized the importance of coordinating the program with the candidate’s dialysis schedules. Several candidates and recipients, their significant others, and healthcare providers also mentioned the importance of involving significant others in the program.

### 3.5 Potential barriers and facilitators

According to the survey data, key factors motivating kidney transplant candidates to work on their overall health were maintaining overall condition (36%), being fit for transplantation (24%), improving wellbeing (23%), and maintaining their overall health status (22%).

During the interviews and focus groups, all participants were asked about potential motivators for kidney transplant candidates to specifically engage in a prehabilitation program. Several key factors emerged as important facilitators (see Table 4).

*“…In the short term, it's manageable without intensive guidance, but to maintain it, especially over a long period, it's helpful to have some form of accountability from time to time." (Kidney transplant candidate 2, focus group 1)*

**Table 4:** Potential facilitators and barriers of prehabilitation.

Facilitators	KTCs, KTRs *N* = 22 (%)	Sig. others *N* = 3 (%)	HC providers *N* = 22 (%)	Barriers	KTCs, KTRs *N* = 22 (%)	Sig. others *N* = 3 (%)	HC providers *N* = 22 (%)
**Emotional, social or practical support** By peers, family or healthcare providers, advice and direction from trained professionals.	**94**	**100**	**50**	**Physical and emotional fatigue** Lack of energy, tiredness, feelings of overwhelm, difficulties concentrating.	**100**	**100**	**50**
**Monitoring and goal setting** Tracking progress, symptoms or behaviors, establishing specific achievable objectives.	**87**	**67**	**18**	**Psychological and behavioral challenges** Symptoms of anxiety and depression, low self-efficacy, emotional distress, low motivation.	**87**	**100**	**59**
**Intrinsic and extrinsic motivation** Internal factors such as personal satisfaction or interest, external factors such as recognition or rewards.	**86**	**100**	**27**	**Geographical and logistical challenges** Long travel distance, transportation issues, scheduling difficulties, limited availability**.**	**80**	**100**	**41**
**Accessibility and convenience** Location proximity, transportation options, minimal travel time, user-friendly programs, availability of remote or home-based options.	**51**	**33**	**41**	**Perceived lack of need** Underestimation of symptoms or risks associated with their condition.	**72**	**33**	**0**
**Knowledge and empowerment** Accurate, relevant information regarding health condition and treatment options, gaining confidence and control in healthcare decisions.	**44**	**67**	**41**	**Physical limitations and comorbidities** Reduced mobility, muscle weakness, comorbidities including diabetes, hypertension and cardiovascular disease	**71**	**0**	**36**
				**Time constraints** Limitations due to various responsibilities, including employment, medical treatment and caregiving duties.	**58**	**33**	**55**
				**Limited health literacy and awareness** Difficulty to understand medical terminology or instructions, unawareness of the importance of lifestyle changes.	**44**	**33**	**32**
				**Financial and socioeconomic challenges** Expenses related to medical treatment, transportation or programs, limited health insurance coverage.	**29**	**33**	**50**
				**Inadequate information provision** Lack of details, use of medical jargon, inconsistent communication.	**22**	**0**	**59**

Note. KTCs: kidney transplant candidates; KTRs: kidney transplant recipients; Sig. Others: significant others kidney transplant candidates and recipients; HC providers: healthcare providers.

Additionally, the survey data highlighted key barriers that candidates face in working on their overall health, primarily due to a perceived sufficiency in health management (32%), as well as symptoms of fatigue (24%), physical impairments (14%), and a lack of motivation or discipline (10%).

During the interviews and focus groups, participants were also asked about potential barriers that could prevent kidney transplant candidates from engaging in a prehabilitation program. Several key factors emerged as important barriers (see Table 4), with the majority aligning with the potential barriers identified in the survey regarding working on overall health.

*“You’ll lose another couple of hours in the afternoon, twice a week. Look, it's half an hour drive, half an hour back. Then you've already lost an hour and when you are there, again three quarters of an hour...Then you're never home anymore." (Kidney transplant candidate, interview 15)*

*"I would quite like work out with supervision once a week to build up more muscle strength but not if I have to pay for it myself, because I just can't afford it financially." (Kidney transplant recipient, interview 2)*

## 4 Discussion

This mixed-methods study explored factors influencing the development and implementation of a prehabilitation program for kidney transplant candidates, guided by the Context and Implementation of Complex Interventions framework. The findings indicate that physical activity receives limited attention in current clinical practice. While nutritional status and psychological well-being are generally addressed, they are often considered primarily upon candidate request rather than as standard components of care. In contrast, dietician-led nutritional management is routinely integrated into standard care for individuals undergoing dialysis. Furthermore, the study highlights that kidney transplant candidates represent a vulnerable group facing a high prevalence of physical, nutritional, and psychological challenges, with nearly half of them experiencing difficulties across multiple domains. There was a strong alignment between the perspectives of kidney transplant candidates and recipients, their significant others, and healthcare providers regarding prehabilitation and its influencing factors. All groups expressed a strong need for prehabilitation, emphasizing that programs should be tailored to individual needs and preferences, implemented in a home-based setting, and guided by a professional. Key facilitators for participation included support and guidance, accessibility and convenience, and social and peer influence. While barriers such as geographical and logistical challenges, perceived lack of need, fatigue, and psychological and behavioral obstacles may hinder engagement.

The qualitative findings showed that physical activity received limited attention in current clinical practice, while quantitative data showed that the majority of candidates reported receiving advice from their healthcare provider to engage in physical activity. This apparent discrepancy may be attributed to the way physical activity is addressed during consultations, as interview and focus group participants noted that it is often mentioned briefly rather than being a central focus of discussion. These findings align with previous research showing that physical activity counseling by nephrologists has historically been infrequent (Delgado and Johansen, 2010). However, Taryana et al. (2019) reported that 47% of nephrologists frequently counseled individuals with kidney failure to increase physical activity levels, while Greenwood et al. (2014) found that 59% of nephrologists inquired about and provided guidance on physical activity. Additionally, our findings confirm prior findings that kidney transplant candidates perceive a lack of physical activity programs or resources within renal care (Taryana et al., 2019). Collectively, these findings underscore the need of incorporating physical activity promotion more consistently into consultations and interventions for this population.

Participants emphasized improvements in strength and endurance as central components for a prehabilitation program, which aligns with existing evidence emphasizing the role of physical activity in managing kidney failure, both for individuals with kidney failure and their significant others (Hemmelgarn et al., 2017; Manns et al., 2014). However, Scheede-Bergdahl et al. (2019) argued that while exercise is often the primary focus in research on prehabilitation, it is crucial to acknowledge the role of nutrition and psychological well-being in both adherence to the program and the overall response to physical training. Evidence showed that psychological interventions before surgery can reduce fatigue levels and symptoms of depression, and improve quality of life in kidney transplant candidates (Barello et al., 2023; Pascoe et al., 2017; Picariello et al., 2017), while malnutrition negatively impacts recovery and overall health (MacLaughlin et al., 2022; McClave et al., 2013; Zha and Qian, 2017). These findings support previous research suggesting that, in addition to physical activity, nutritional status and psychological well-being are important components to consider within prehabilitation programs (Cardol et al., 2022; Carli and Scheede-Bergdahl, 2015; Scheede-Bergdahl et al., 2019). The variability in health status among kidney transplant candidates, with challenges occurring in one or more domains, suggests that a singular approach to prehabilitation may not be effective and that a tailored, multimodal program is essential to meet the diverse needs of this population.

In line with existing literature, participants also expressed the importance of a home-based intervention and guidance by a professional (Baker et al., 2022; Coyne et al., 2023; Hayden et al., 2024; Ma et al., 2022; Young et al., 2024). Additionally, the study findings showed a high variation in perceived barriers and motivators for engaging in a prehabilitation program among individuals with kidney failure. This is consistent with other studies highlighting the complexity and diversity of factors that influence engagement in healthy lifestyle behaviors (Cardol et al., 2022; Clarke et al., 2015; Hannan and Bronas, 2017; Young et al., 2024). Prior research showed that both the persons’ physical and social environment play an important role in supporting lifestyle (Cardol et al., 2022; Chen et al., 2018; Evangelidis et al., 2019). Burton et al. (2018) suggested that involving peers can promote and maintain adherence to exercise programs. These insights suggest that prehabilitation should not be viewed solely as an individual effort but rather as a combined initiative. Moreover, the role of social influence highlights the potential benefits of group-based interventions and underscores the importance of incorporating social and environmental components in the development of effective interventions.

This study has several strengths. To our knowledge, this is the first mixed-methods study to explore factors that are of influence on the development and implementation of a multimodal prehabilitation program for kidney transplant candidates. The use of the Context and Implementation of Complex Interventions framework enabled the systematic exploration of contextual factors that influence the development and implementation of prehabilitation intervention in clinical practice. The use of a mixed-methods approach also enabled comprehensive data collection and analysis, offering deeper insights into the complexities of prehabilitation. By incorporating perspectives from kidney transplant candidates and recipients, their significant others, and healthcare providers across multiple hospitals in the Northern Netherlands, this study ensures that prehabilitation recommendations align with the actual needs, preferences, and realistic goals of kidney transplant candidates within existing care structures. Furthermore, the study highlights a multimodal approach to prehabilitation, addressing not only physical activity but also other important lifestyle components essential for optimizing patient outcomes. This person-centered approach supports sustainable behavior change and could enhance the likelihood of candidates maintaining healthy lifestyle behaviors after completing a prehabilitation program, although this remains to be confirmed in future research. However, some limitations should be acknowledged. First, COVID-19 restrictions prevented in-person interviews and focus groups, which could have affected the depth of understanding of participants’ situations and perspectives. Second, potential selection bias may have influenced the findings, as participants who were more interested in prehabilitation or in better overall health may have been more likely to participate. Third, the generalizability of the findings may be limited, as the study was conducted in the northern region of the Netherlands, where the population may differ from that of other regions. Lastly, reliance on self-reported data introduces the potential for personal biases or perceptions to influence the results.

## 5 Conclusions

This study provides valuable insights into the factors influencing the development and implementation of a prehabilitation program for kidney transplant candidates, emphasizing the importance of a structured, person-centered, and multimodal approach. The findings highlight gaps in current clinical practice, including the limited attention given to physical activity and a reactive, rather than proactive, approach to nutritional and psychological support. Given the high prevalence of physical, nutritional, and psychological problems in this population, a tailored prehabilitation program aligned with a person needs and preferences is essential. Kidney transplant candidates and recipients, their significant others, and healthcare providers all recognize the value of prehabilitation, particularly when it involves a home-based, professionally guided program tailored to individual circumstances. Key facilitators such as support, guidance, accessibility, convenience, and social influence play an important role in encouraging participation in a prehabilitation program. However, addressing barriers such as logistical constraints, fatigue, and perceived lack of necessity is crucial for optimizing engagement. Future research should focus on the development of evidence-based, theory-informed prehabilitation interventions that improve both pre- and post-transplant outcomes for individuals with kidney failure in which these barriers are taken into account. The insights of our study can be used as the foundation for a person-centered, multimodal prehabilitation program tailored to the needs of kidney transplant candidates.

## Acknowledgements

The authors sincerely thank all participants for contributing their time, effort, and valuable insights to this study.

## Funding sources

This paper was funded by the Dutch Kidney Foundation (grant number 20OS008).

## Study registration

This study was approved by the Medical Ethical Committee of the UMCG (METc 2021/461). Registration date: 3-8-2021. Start of recruitment: 4 November 2021.

## Data availability statement

The data that support the findings of this study are stored on a secured drive of the PreCareTx project at the University Medical Center Groningen. Due to the presence of identifiable information in the interview and focus group recordings, the raw data cannot be shared publicly. De-identified data may be made available from the corresponding author upon reasonable request.

## CRediT authorship contribution statement

**Avril J. Haanstra:** Writing – review & editing, Writing – original draft, Visualization, Project administration, Methodology, Investigation, Formal analysis, Data curation, Conceptualization. **Yvonne van der Veen:** Writing – review & editing. **Heleen Maring:** Writing – review & editing. **Evelien E. Quint:** Writing – review & editing, Conceptualization. **Adelita V. Ranchor:** Writing – review & editing, Supervision. **Stefan P. Berger:** Writing – review & editing, Supervision. **Stephan J.L. Bakker:** Writing – review & editing. **Evelyn J. Finnema:** Writing – review & editing, Supervision. **Coby Annema:** Writing – review & editing, Visualization, Supervision, Resources, Project administration, Methodology, Investigation, Funding acquisition, Formal analysis, Conceptualization.

## Declaration of competing interest

The authors declare the following financial interests which may be considered as potential competing interests: Author Coby Annema reports financial support was provided by the Dutch Kidney Foundation. The other authors declare that they have no known competing financial interests or personal relationships that could have appeared to influence the work reported in this paper.

## Appendix Supplementary materials

Image, application 1
