# Supplementary material for: Factors influencing the development and implementation of a prehabilitation program for kidney transplant candidates: A mixed-methods contextual analysis
Source: Int J Nurs Stud Adv. 2026 May 18;10:100558. doi: 10.1016/j.ijnsa.2026.100558 (PMC13202596; doi:10.1016/j.ijnsa.2026.100558)
Supplement: Supplementary file 1 [file mmc1.docx]

# **Supplementary material 1**

## *Semi-structured interview guide kidney transplant candidates*

1. What is your age?
2. What is your marital status?
3. What is the highest level of education you have completed?
4. Are you currently employed?
   1. If yes, what type of work do you do?
   2. If not, what is the reason you stopped working?
   3. If not, how long have you been out of work?
5. When were you diagnosed with kidney disease?
6. Are you currently on dialysis?
   1. Duration
   2. Dialysis method
7. *Administer DASI*
8. Can you tell me something about your physical functioning, nutritional status and psychological well-being?
   1. Physical functioning
      1. Sport/exercise
      2. Household activities
      3. Work
      4. Strength
      5. Endurance
   2. Nutritional status
      1. Eating patterns
      2. Diet
      3. Appetite
      4. Weight
   3. Psychological well-being
      1. Stress (worrying)
      2. Fatigue
      3. Sleep
      4. Anxiety
      5. Depression
      6. Tension/relaxation
9. What do you do yourself to improve your overall health?
   1. Yes: how do you do this?
   2. Yes: how is it going?
      1. Positive/negative experiences
   3. If yes, what is the reason for working on your overall health?
   4. If not, can you explain why you are not working on your overall health?
   5. If not, what is holding you back from doing so?
10. Do you receive support or has support been offered to you to work on your overall health?
    1. If yes, from whom do you receive support?
       1. Primary care
       2. General practitioner
       3. Hospital
    2. If yes, what kind of support do you receive?
       1. Course/program
       2. Training
       3. Diet
    3. If yes, what did the support look like?
       1. Who
       2. Frequency
       3. Structure
    4. If yes, do you feel that you receive enough support, and why?
    5. If not do you feel you need support?
       1. If yes, what kind of support do you need?
          1. Who, what type of support
       2. If not, what is the reason you don’t feel the need for support?

1. What do you feel is currently missing in the care regarding lifestyle (physical functioning, nutritional status, psychological well-being)?
   1. How would you structure the care to improve the well-being of kidney transplant candidates?
   2. How should the support in the program be structured to be best prepare you for the transplant?
      1. Program structure
      2. Group, individual, or combined participation
      3. Location
      4. Guidance
   3. What advice do you have regarding structuring care to improve the overall health of kidney transplant candidates?
      1. Barriers/facilitators
   4. Do you have any additional comments that you think are important to improve the overall health of kidney transplant candidates?

## *Semi-structured interview guide kidney transplant candidates (Dutch)*

1. Wat is uw leeftijd?
2. Wat is uw burgerlijke staat?
3. Wat is de hoogste opleiding die u heeft afgerond?
4. Werkt u op dit moment?
   1. Ja: wat voor werk doet u?
   2. Nee: wat is de reden dat u gestopt bent met werken?
   3. Nee: hoe lang bent u gestopt met werken?
5. Wanneer heeft u de diagnose van uw nierziekte gekregen?
6. Dialyseert u op dit moment?
   1. Tijdperiode
   2. Dialyse methode
7. *DASI vragenlijst afnemen*
8. Kunt u iets vertellen over uw lichamelijk functioneren, voedingstoestand and psychologisch welzijn?
   1. Lichamelijk functioneren
      1. Sport
      2. Huishouden
      3. Werk
      4. Kracht
      5. Uithoudingsvermogen
   2. Voedingstoestand
      1. Voedingspatroon
      2. Dieet
      3. Eetlust
      4. Gewicht
   3. Psychologisch welzijn
      1. Stress (zorgen maken)
      2. Vermoeidheid
      3. Slaap
      4. Angst
      5. Depressie
      6. (Ont)spanning
9. Wat doet u zelf om uw conditie te verbeteren?
   1. Ja: hoe doet u dit?
   2. Ja: hoe gaat dat?
      1. Positieve/negatieve ervaringen
   3. Ja: wat is de reden dat u aan uw conditie werkt?
   4. Nee: kunt u vertellen wat de reden is dat u niet aan uw conditie werkt?
   5. Nee: wat houdt u tegen?
10. Ontvangt u hulp of is u hulp aangeboden bij het werken aan uw conditie?
    1. Ja: van wie ontvangt u hulp?
       1. Eerste lijn
       2. Huisarts
       3. Ziekenhuis
    2. Ja: wat voor hulp ontvangt u?
       1. Cursus/programma
       2. Training
       3. Dieet
    3. Ja: hoe zag de begeleiding eruit?
       1. Wie
       2. Hoe vaak
       3. Opzet
    4. Ja: vindt u dat u voldoende steun krijgt en wat is de reden dat u dat vindt?
    5. Nee: heeft u hier wel behoefte aan?
       1. Ja: waar heeft u behoefte aan?
          1. Wie, wat voor hulp
       2. Nee: wat is de reden dat u hier geen behoefte aan heeft?

1. Wat mist u op dit moment in de zorg met betrekking tot leefstijl (lichamelijke functioneren, voedingstoestand, psychologische welzijn) voor transplantatie?
   1. Hoe zou u de zorg willen vormgeven om de conditie te verbeteren van niertransplantatie kandidaten?
   2. Hoe zou voor u de ondersteuning in het programma eruit moeten zien om u zo goed mogelijk voor te bereiden op de transplantatie?
      1. Programma opzet
      2. Groep, individueel of combinatie
      3. Locatie
      4. Begeleiding
   3. Welke adviezen heeft u bij het vormgeven van de zorg om de conditie te verbeteren van niertransplantatie kandidaten?
      1. Belemmerende, bevorderen factoren
   4. Heeft u nog op- of aanmerkingen die u wilt delen dat van belang kan zijn om de conditie van niertransplantatie kandidaten te verbeteren?

# **Supplementary material 2**

## *Semi-structured interview guide kidney transplant recipients*

1. What is your age?
2. What is your marital status?
3. What is the highest level of education you have completed?
4. What kind of work did you do before your transplant?
5. When were you diagnosed with kidney disease?
6. Did you undergo dialysis before the transplant?
   1. Duration
   2. Dialysis method
7. When did you receive your kidney transplant?
8. *Administer DASI*
9. Can you tell me something about your physical functioning, nutritional status and psychological well-being before transplant?
   1. Physical functioning
      1. Sport/exercise
      2. Household activities
      3. Work
      4. Strength
      5. Endurance
   2. Nutritional status
      1. Eating patterns
      2. Diet
      3. Appetite
      4. Weight
   3. Psychological well-being
      1. Stress (worrying)
      2. Fatigue
      3. Sleep
      4. Anxiety
      5. Depression
      6. Tension/relaxation
10. What did you do yourself to improve your overall health before transplant?
    1. If yes, how did you do this?
    2. If yes, how did it go?
       1. Positive/negative experiences
    3. If yes, what was the reason for working on your overall health?
    4. If not, can you explain why you did not work on your overall health?
    5. If not, what was holding you back from doing so?
11. Did you receive support or was support offered to you to work on your overall health?
    1. If yes, from whom did you receive support?
       1. Primary care
       2. General practitioner
       3. Hospital
    2. If yes, what kind of support did you receive?
       1. Course/program
       2. Training
       3. Diet
    3. If yes, what did the support look like?
       1. Who
       2. Frequency
       3. Structure
    4. If yes, did you feel you received enough support, and why?
    5. If not, did you feel you needed support?
       1. If yes, what kind of support did you need?
          1. Who, what type of support
       2. If not, what is the reason you didn’t felt the need for support?
12. What did you feel was missing in the care regarding lifestyle (physical functioning, nutritional status, psychological well-being)?
    1. How would you have structured the care to improve the well-being of kidney transplant candidates?
    2. How should the support in the program have been structured to be best prepare you for the transplant?
       1. Program structure
       2. Group, individual, or combined participation
       3. Location
       4. Guidance
    3. What advice do you have regarding structuring care to improve the overall health of kidney transplant candidates?
       1. Barriers/facilitators
    4. Do you have any additional comments that you think are important to improve the overall health of kidney transplant candidates?

## *Semi-structured interview guide kidney transplant recipients (Dutch)*

1. Wat is uw leeftijd?
2. Wat is uw burgerlijke staat?
3. Wat is de hoogste opleiding die u heeft afgerond?
4. Wat voor werk deed u voor transplantatie?
5. Wanneer heeft u de diagnose van uw nierziekte gekregen?
6. Heeft u gedialyseerd voor transplantatie?
   1. Tijdperiode
   2. Dialyse methode
7. Wanneer bent u getransplanteerd?
8. *DASI vragenlijst afnemen*
9. Kunt u iets vertellen over uw lichamelijke en mentale conditie voor transplantatie?
   1. Lichamelijke conditie
      1. Sport
      2. Huishouden
      3. Werk
      4. Kracht
      5. Uithoudingsvermogen
   2. Voedingstoestand
      1. Voedingspatroon
      2. Dieet
      3. Eetlust
      4. Gewicht
   3. Mentale conditie
      1. Stress (zorgen maken)
      2. Vermoeidheid
      3. Slaap
      4. Angst
      5. Depressie
      6. (Ont)spanning
10. Wat heeft u zelf gedaan om uw conditie te verbeteren?
    1. Ja: hoe deed u dit?
    2. Ja: hoe ging dat?
       1. Positieve/negatieve ervaringen
    3. Ja: wat was de reden dat u aan uw conditie heeft gewerkt?
    4. Nee: kunt u vertellen wat de reden was dat u niet aan uw conditie heeft gewerkt?
    5. Nee: wat hield u tegen?
11. Ontving u hulp of is u hulp aangeboden bij het werken aan uw conditie?
    1. Ja: van wie heeft u hulp ontvangen?
       1. Eerste lijn
       2. Huisarts
       3. Ziekenhuis
    2. Ja: wat voor hulp heeft u ontvangen?
       1. Cursus/programma
       2. Training
       3. Dieet
    3. Ja: hoe zag de begeleiding eruit?
       1. Wie
       2. Hoe vaak
       3. Opzet
    4. Ja: vond u dat u voldoende steun heeft gekregen en wat is de reden dat u dat vindt?
    5. Nee: had u hier wel behoefte aan?
       1. Ja: waar had u behoefte aan?
          1. Wie, wat voor hulp
       2. Nee: wat is de reden dat u hier geen behoefte aan had?

1. Wat heeft u gemist in de zorg met betrekking tot leefstijl (lichamelijke conditie, voedingstoestand, mentale conditie) voor transplantatie?
   1. Hoe zou u de zorg willen vormgeven om de conditie te verbeteren van niertransplantatie kandidaten?
   2. Hoe zou voor u de ondersteuning in het programma eruit moeten hebben gezien om u zo goed mogelijk voorbereid te hebben op de transplantatie?
      1. Programma opzet
      2. Groep, individueel of combinatie
      3. Locatie
      4. Begeleiding
   3. Welke adviezen heeft u bij het vormgeven van de zorg om de conditie te verbeteren van niertransplantatie kandidaten?
      1. Belemmerende, bevorderen factoren
   4. Heeft u nog op- of aanmerkingen die u wilt delen dat van belang kan zijn om de conditie van niertransplantatie kandidaten te verbeteren?

# **Supplementary material 3**

## *Semi-structured interview guide medical specialists*

1. Hospital: ………………………………………………………………….
2. Role/Position: nephrologist/nurse specialist/……………………………..
3. How many years have you been working as a nephrologist / nurse practitioner in nephrology?
   ..…. years
4. Approximately how many adult patients are being treated for a kidney disease in your hospital? _____________ patients
5. How often do kidney patients on the transplant waiting list visit your outpatient clinic? / How is care currently organized for patients on the kidney transplant waiting list? For example, how many outpatient appointments are usually scheduled per year for kidney patients in your hospital?
   - 0-1 time per month
   - 1 time per 2 months
   - Every 3 months
   - Every 4 months
   - Every 6 months
   - Once per year
   - ……………………………………………..
6. What topics are routinely discussed during a consultation (besides kidney disease, medication etc.)? For example, is lifestyle addressed? / During an outpatient consultation, is attention routinely given to:

|  | Yes | No |
| --- | --- | --- |
| Physical activity (intensity of moderate/vigorous activities) |  |  |
| Following a diet |  |  |
| Smoking/quit smoking |  |  |
| Alcohol consumption |  |  |
| Stress or psychosocial problems related to kidney disease |  |  |

1. Which healthcare providers, besides medical specialists, are routinely involved in the care for kidney patients in your center, and what is their role?
2. Which healthcare providers could be involved if we implement a prehabilitation program for kidney patients? And in what way?

|  | Routinely involved | To involve in the PreCareTx program? |
| --- | --- | --- |
| Dietitian |  |  |
| Physiotherapist |  |  |
| Social worker |  |  |
| Nurse specialist |  |  |
| Psychologist |  |  |
| Other: ……………………………………… |  |  |

1. What lifestyle-related care (nutrition, physical activity, psychosocial support) is currently offered to kidney patients (outside the outpatient consultation)? For example, programs or courses? How is this care structured?
2. Offered program/course/diet
3. Guidance
4. Advice
5. Which healthcare providers are involved
6. In your opinion, how should a prehabilitation program for kidney transplant candidates be structured, and can you explain why? *Considering the patients you see at the clinic, are there specific aspects we should take into account when designing the program?*
   1. Individual vs group vs combination participation
   2. Guidance/by whom
   3. During dialysis or not
   4. Physical activity
   5. Nutrition
   6. Learning to cope with …
7. What do you think are potential facilitating factors? What could motivate patients to participate in prehabilitation? How could we best implement this?
8. What do you think are potential barriers to providing prehabilitation to this group? What should we take into account when designing the program? How could we best address this?
9. Finances
10. Distance (group sessions)
11. Time
12. Comorbidities
13. Fatigue
14. Environment (physical/social)
15. How could we best involve you, as a healthcare provider, in the prehabilitation program? How would you see your role in supporting it, and what would you need to carry out that role?
16. Involving patients (recruitment)
17. Addressing it during consultations (introducing the program/motivating participation/monitoring progress)
18. Which other healthcare professionals do you think we should also speak with?
19. Nurse specialist
20. Dietitian
21. Social worker
22. Physiotherapist

## *Semi-structured interview guide medical specialists (Dutch)*

1. Ziekenhuis: ………………………………………………………………….
2. Functie: nefroloog/verpleegkundig specialist/…………………………..…..
3. Hoeveel jaar bent u werkzaam als nefroloog/verpleegkundig specialist nefrologie?
   ..…. jaar
4. Hoeveel volwassen patiënten zijn (bij benadering) in behandeling voor een nieraandoening in uw ziekenhuis? _____________ patiënten
5. Hoe vaak komen nierpatiënten die op de wachtlijst staan voor niertransplantatie bij jullie op de poli? / Hoe verloopt nu de zorg voor patiënt die op de wachtlijst staan voor een niertransplantatie? Bv. Hoe vaak per jaar worden normaal gesproken poliklinische afspraken gepland voor nierpatiënten in uw ziekenhuis?
   - 0-1 keer per maand
   - 1 keer per 2 maand
   - Om de 3 maanden
   - Om de 4 maanden
   - Om de 6 maanden
   - 1 keer per jaar
   - ……………………………………………..
6. Wat wordt **standaard** besproken in een consult (naast bv. de nieraandoening, medicatie e.d.), is er bijv. ook aandacht voor leefstijl: / Wordt in een poliklinisch consult **standaard aandacht** besteedt aan:

|  | Ja | Nee |
| --- | --- | --- |
| Lichaamsbeweging (mate van inspannende/matig inspannende activiteiten) |  |  |
| Het opvolgen van het dieet |  |  |
| Roken/stoppen met roken |  |  |
| Alcoholgebruik |  |  |
| Stress of psychosociale problemen t.g.v. de nieraandoening |  |  |

1. Welke hulpverleners zijn naast medisch specialisten standaard betrokken bij de zorg voor nierpatiënten in uw centrum en wat is hun rol daarin?
2. Welke hulpverleners kunnen wij betrekken wanneer we het prehabilitatie programma gaan aanbieden aan de patiënt? En op welke manier?

|  | Standaard betrokken | Betrekken  Interventie  PreCareTx? |
| --- | --- | --- |
| Diëtiste |  |  |
| Fysiotherapeut |  |  |
| Maatschappelijk werk |  |  |
| Verpleegkundig specialist |  |  |
| Psycholoog |  |  |
| Anders: ……………………………………… |  |  |

1. Welke zorg op het gebied van leefstijl verandering (voeding, beweging, en psychosociaal) wordt er momenteel aangeboden aan nierpatiënten (los van het poliklinisch consult)? Zijn er bv. aangeboden programma’s of cursussen? En hoe is deze zorg vormgegeven?
2. Aangeboden programma/cursus/dieet
3. Begeleiding
4. Advies
5. Welke hulpverleners zijn hierbij betrokken
6. Hoe zou een prehabilitatie programma voor nierpatiënten volgens u vormgegeven moeten worden en kunt u uw keuze toelichten?

*Als u kijkt naar de patiënten die u ziet op de poli, zijn er bepaalde aspecten volgens u waar wij rekening mee moeten houden bij het vormgeven van het programma?*

- 1. Individueel vs groep vs combinatie
  2. Begeleiding? Door wie
  3. Wel of niet tijdens dialyse
  4. Bewegen
  5. Voeding
  6. Leren omgaan met …

1. Wat zijn volgens u mogelijke bevorderende factoren, wat zou voor de patiënten stimulerend werken om deel te nemen aan prehabilitatie? Hoe kunnen we dit het beste doen?
2. Wat zijn volgens u mogelijke belemmeringen om prehabilitatie aan deze groep te kunnen geven? Waar moeten we rekening mee houden bij het vormgeven? Hoe kunnen we dit het beste doen?
3. Financiën
4. Afstand (groepsles)
5. Tijd
6. Comorbiditeiten
7. Vermoeidheid
8. Omgeving (fysiek/sociaal)
9. Hoe zouden we u als zorgverlener het beste kunnen betrekken bij het prehabilitatieprogramma? Hoe ziet u deze rol bij eventuele ondersteuning voor zich? En wat heeft u daarvoor nodig?
10. Includeren patiënten (rekruteren)
11. Aandacht in consult (onder de aandacht brengen/motiveren om mee te doen/voortgang bij deelname)
12. Met welke betrokken hulpverleners zouden wij ook nog in gesprek moeten volgens u?
13. Verpleegkundig specialist
14. Diëtist
15. Maatschappelijk werker
16. Fysio

# **Supplementary material 4**

## *Semi-structed focus group guide kidney transplant candidates*

1. Is the situation described familiar to you?
   1. What challenges do you encounter most?
   2. Are you currently receiving any support for this?
   3. If yes, what kind of support?
   4. If not, do you feel a need for support? If so, what kind?
2. How do you think a prehabilitation program for kidney patients should be designed, and could you explain your reasoning?
   1. Individual group or a combination?
   2. Guidance? By whom?
   3. Should it take place during dialysis or not?
   4. Should the significant others be involved?
3. In your opinion, what should be the main focus when preparing for a kidney transplant?
4. Could you rank these three components from most to least important, and explain your reasoning? Can you also write down how you envision this? Wilt u ook opschrijven hoe u dit voor u ziet?
   1. Physical activity (strength/endurance/flexibility)
   2. Nutrition (healthy weight/optimizing nutritional deficiencies)
   3. Coping skills (managing stress/fatigue)
5. What would help or motivate you to participate in a prehabilitation program? What do you think would work for you, and how could we make that happen?
   1. Guidance
   2. Monitoring
   3. Peer-to-peer support
   4. Involve significant others
6. What might prevent or hinder you from participating in a prehabilitation program? How could we address or reduce these barriers?
7. Fatigue
8. Financial constrains
9. Distance (for group sessions)
10. Time
11. Comorbidities

## *Semi-structed focus group guide kidney transplant candidates (Dutch)*

1. Is deze geschetste problematiek herkenbaar?
   1. Waar loopt u zelf het meeste tegen aan?
   2. Ontvangt u daar momenteel hulp bij?
   3. Zo ja, welke hulp?
   4. Zo nee, wel behoefte aan hulp? Zo ja, welke hulp?
2. Hoe zou een prehabilitatie programma voor nierpatiënten volgens u opgezet moeten worden en kunt u uw keuze toelichten?
   1. Individueel vs groep vs combinatie?
   2. Begeleiding? Door wie?
   3. Wel of niet tijdens dialyse?
   4. Omgeving betrekken?
3. Waar moet volgens u de meeste aandacht naar uit gaan ter voorbereiding op de niertransplantatie?
4. Kunt u voor uzelf de drie onderdelen rangschikken van de meeste aandacht naar minder aandacht en kunt u uw keuze daarbij toelichten: waarom vindt u dat? Wilt u ook opschrijven hoe u dit voor u ziet?
   1. Bewegen (kracht/conditie/flexibiliteit)
   2. Voedingstoestand (gezond gewicht/voedingstekorten opheffen)
   3. Leren omgaan met (stress/vermoeidheid)
5. Wat zou voor u behulpzaam of stimulerend zijn om mee te doen aan een prehabilitatieprogramma. Wat zou voor u werken? Hoe kunnen we dat bewerkstelligen?
   1. Ondersteuning
   2. Monitoring
   3. Lotgenotencontact
   4. Omgeving betrekken
6. Wat zou u tegenhouden of belemmeren om mee te doen aan het prehabilitatieprogramma? Hoe kunnen we dit voorkomen/beïnvloeden?
7. Vermoeidheid
8. Financiën
9. Afstand (groepsles)
10. Tijd
11. Comorbiditeiten

# **Supplementary material 5**

## *Semi-structed focus group guide significant others kidney transplant candidates*

1. Is the situation described familiar to you? Do you see recognize this in your partner of family member?
   1. What challenges does your partner of family member face most often?
   2. Are they currently receiving any support for this?
      1. If yes, what kind of support?
      2. If not, do they feel a need for support? If so, what kind?
   3. What challenges do you personally face most often?
   4. Are you currently receiving any support for this?
      1. If yes, what kind of support?
      2. If not, do you feel a need for support>? If so, what kind?
2. How do you think a prehabilitation program for kidney patients should be designed, and could you explain your reasoning?
   1. Individual group or a combination?
   2. Guidance? By whom?
   3. Should it take place during dialysis or not?
   4. Physical activity
   5. Nutrition
   6. Learning to cope with …
3. How do you think the patient’s environment could be best involved in the prehabilitation program? How would you see your own role in supporting it, and what would you need to do this effectively?
   1. Emotional support
   2. Encouraging healthy behaviors
   3. Transportation (e.g., group sessions)
4. What might prevent or make it difficult for you to provide support to your partner or family member?
   1. Financial constraints
   2. Distance
   3. Time
   4. Health condition(s)

## *Semi-structed focus group guide significant others kidney transplant candidates (Dutch)*

1. Is deze geschetste problematiek herkenbaar? Ziet u dit terug bij uw partner/familielid?
   1. Waar loopt uw partner of familielid zelf het meeste tegen aan?
   2. Ontvangt hij of zij daar momenteel hulp bij?
   3. Zo ja, welke hulp?
   4. Zo nee, heeft hij of zij wel behoefte aan hulp? Zo ja, welke hulp?
   5. Waar loopt uzelf het meeste tegen aan?
   6. Ontvangt u daar momenteel hulp bij?
   7. Zo ja, welke hulp?
   8. Zo nee, heeft u wel behoefte aan hulp? Zo ja, welke hulp?
2. Hoe zou een prehabilitatie programma voor nierpatiënten volgens u opgezet moeten worden en kunt u uw keuze toelichten?
   1. Individueel vs groep vs combinatie?
   2. Begeleiding? Door wie?
   3. Wel of niet tijdens dialyse?
   4. Bewegen
   5. Voeding
   6. Leren omgaan met ..
3. Hoe zouden we de omgeving het beste kunnen betrekken bij het prehabilitatie programma? Hoe ziet u uw rol bij eventuele ondersteuning voor zich? En wat heeft u hierbij nodig?
   1. Mentale steun
   2. Stimuleren gedrag
   3. Vervoer (afstand groepsles)
4. Wat zou u tegen houden of belemmeren om steun te geven aan uw familielid of partner?
   1. Financiën
   2. Afstand
   3. Tijd
   4. Aandoening(en)

# **Supplementary material 6**

## *Semi-structed focus group guide healthcare providers*

1. Can you describe how you are currently involved in the care of kidney patients during the waitlist period, and what this care entails?

a. Frequency of appointments
b. Content of the consultations
c. Involved by whom (referring physician, patient themselves)

1. In your view, what factors could facilitate the implementation of a prehabilitation program for kidney patients? Based on your experience, what would motivate patients to participate in such a program, and how could we best achieve this?
2. What do you think could make it difficult to implement a prehabilitation program for kidney patients? What should we consider when designing the program, and how could we best address these challenges?
   1. Financial constraints
   2. Distance (for group sessions)
   3. Time
   4. Comorbidities
   5. Fatigue
   6. Environment (physical/social)
3. How do you think healthcare providers could be best involved in the prehabilitation program? What would your role look like, and what support would you need to carry it out?

## *Semi-structed focus group guide healthcare providers (Dutch)*

1. Kunnen jullie vertellen hoe jullie nu betrokken zijn bij de zorg voor nierpatiënten gedurende de wachtlijst periode en waar deze zorg uit bestaat?

a. Frequentie van afspraken
b. Inhoud van het consult
c. Door wie betrokken (verwijzend arts/patiënt zelf)

1. Wat zijn volgens jullie mogelijke bevorderende factoren bij het invoeren van een prehabilitatie programma voor nierpatiënten? Op basis van jullie ervaring; wat zou voor patiënten stimulerend werken om deel te nemen aan een prehabilitatie programma? Hoe kunnen we dit het beste doen?
2. Wat zijn volgens jullie mogelijke belemmeringen bij het invoeren van een prehabilitatie programma voor nierpatiënten? Waar moeten we rekening mee houden bij het vormgeven? Hoe kunnen we dit het beste doen?
   1. Financiën
   2. Afstand (groepsles)
   3. Tijd
   4. Comorbiditeiten
   5. Vermoeidheid
   6. Omgeving (fysiek/sociaal)
3. Hoe zouden we jullie als zorgverleners het beste kunnen betrekken bij het prehabilitatieprogramma? Hoe zou deze rol er uit kunnen zien? Wat hebben jullie daarvoor nodig?
